# Exploring Trends and Sentiments in Epilepsy Discussions: A Thematic Analysis of the r/Epilepsy Subreddit (2023–2024)

**DOI:** 10.3390/neurolint18030047

**Published:** 2026-03-01

**Authors:** Kelly Fisher, Eliza Sejdiu, Michelle You, Rahim Hirani, Adam Karp, Mill Etienne

**Affiliations:** 1 School of Medicine, New York Medical College, Valhalla, NY 10595, USA; 2 Department of Neurology, New York Medical College, Valhalla, NY 10595, USA

**Keywords:** epilepsy, social media, reddit, sentiment analysis, natural language processing

## Abstract

**Background:** In 2024, Reddit, an emerging social media platform, saw a 50% increase in monthly users to nearly 100 million. Reddit has also emerged as a significant space for discussions about health conditions, including epilepsy, which affects about 50 million people globally. **Purpose:** This study aims to explore trends in the volume, timing, themes, emotional tone, and sentiment of posts on the r/Epilepsy subreddit from 1 December 2023 to 31 December 2024. **Methods:** We collected 25,222 original English-language posts from r/Epilepsy using Reddit’s Application Programming Interface (API). Data extraction was restricted to English-language submissions to ensure compatibility with sentiment and thematic analyses. We analyzed post volume and timing using chi-square tests and Poisson regression. Emotional tone was measured using TextBlob (version 0.19.0), while compound sentiment scores were calculated via VADER (Valence Aware Dictionary and Sentiment Reasoner) (NLTK version 3.9.1). A Pearson correlation assessed agreement between sentiment and emotional tone, with statistical significance set at *p* < 0.05. Thematic analysis was conducted using a KMeans clustering algorithm (scikit-learn version 1.6.1) to identify recurring discussion topics. **Results:** Total monthly posts steadily increased, with the highest number (2175) in December 2024. Peak posts in descending order were in December 2024, August 2024, and November 2024. Posts were not evenly distributed across the week, with a significant peak on Mondays (χ^2^ = 86.75, *p* < 0.001) and Poisson regression confirming higher activity early in the week (*p* = 0.001). Emotional tones fluctuated, with positive sentiments in January and October 2024, and negative sentiments in March and August 2024. KMeans clustering identified five main themes: treatment experiences, community engagement, personal experiences, solidarity, and subreddit gratitude. Manual validation of a random subset of posts demonstrated moderate concordance between automated sentiment classification and human ratings. **Conclusions:** This study highlights temporal patterns, sentiment dynamics, and thematic structure in online discussions on epilepsy. Social media may offer valuable, real-time insights into patient-centered concerns and community engagement, which can inform healthcare professionals and advocacy groups in supporting individuals affected by epilepsy. Future studies may compare trends of epilepsy discussions across various social media platforms, such as X and Instagram, to further understand online patient experiences.

## 1. Introduction

As social media platforms have evolved and expanded over time, social media usage has adapted and increased significantly. As of 2024, more than 5.04 billion people worldwide actively use social media, representing over 62% of the global population [1]. Social media has been an essential tool for individuals seeking health information, medical advice, and connections with patients and communities of various health conditions. In 2024, a survey conducted by Healthline and YouGov found that more than half of United States adults turn to social media for health guidance [2]. Patients increasingly turn to social media for medical advice and emotional support, making it a critical tool for patient engagement and an important area of research and expansion for health care professionals [3].

In 2024, Reddit, an emerging social media platform, saw a 50% increase in monthly users to nearly 100 million [4]. Topic-specific communities, called “subreddits,” have been expanding to provide spaces for specific communities of individuals to share, discuss, and explore health information [5]. Reddit has emerged as a significant space for discussions about health conditions, where users can openly share their personal experiences with medical conditions and healthcare systems, while also engaging with others’ posts on their experiences and insights on similar health challenges. Compared to other platforms such as Facebook or Instagram, Reddit offers unique affordances for health-related discussions. Its structure supports long-form, anonymous conversations that are not algorithmically sorted by popularity or engagement, enabling more nuanced peer-to-peer exchanges. This makes it particularly valuable for understanding authentic, patient-generated discourse around stigmatized or complex conditions such as epilepsy, where anonymity may encourage greater openness.

Epilepsy is a chronic neurological disorder characterized by recurrent, unprovoked seizures due to abnormal brain electrical activity, with significant neurobiological, cognitive, psychological, and social consequences [6]. Epilepsy is one of the most common neurological diseases, affecting about 50 million people globally. Overall, 80% of individuals with epilepsy live in low- and middle-income countries, where clinical diagnoses and medical treatments may be limited or delayed [7]. The internet and social media platforms may offer a bridge to medical support and communities of individuals with similar medical conditions. Engagement in online patient communities has been shown to improve self-management among individuals with epilepsy. A study involving U.S. military veterans demonstrated that participation in an internet-based psychosocial online community led to significant improvements in epilepsy self-management and self-efficacy scores, with the highest improvement specifically in medication adherence and tracking seizure events [8].

Individuals’ participation in online communities and social media platforms has been shown to reduce feelings of isolation, as social media platforms can offer connections to others going through similar challenges [9]. Social support and community can be essential for chronic diseases like epilepsy, where social isolation and stigmatization can be common. Internet-based communities may offer an accessible and low-cost opportunity for support, especially for individuals with limited access to in-person medical care or epilepsy specialists [9]. Social media platforms may also provide patients with insights into new potential therapies or management techniques that may not be commonly offered in their local clinical facilities [10].

Although there have been many investigations on social media platforms and the influence and discussions of various health conditions, there is relatively limited research that is focused specifically on social media postings and discussions, specifically on epilepsy. This highlights the importance of a more targeted investigation into the influence and trends in social media in the epilepsy community. A netnographic study of conversations on epilepsy across social media sites, ranging from foundations, public health organizations, and message boards, was conducted over 12 months in 2018 to 2019 in Europe, and identified key themes and concerns of patients and caregivers. Some key themes discovered across social media sites included epilepsy awareness, psychological and physical impacts of the disease, and treatment and side effect management [11]. Popoola-Samuel et al. conducted an analysis of Instagram posts containing hashtags related to epilepsy, expanding into an investigation of the accuracy and reliability of content. This has become a major area of concern in the era of user-generated health content. They found that over 76% of epilepsy-related Instagram posts collected in 2022 contained true information, but nearly one in four posts contained false or misleading information. This finding raises important concerns about the potential for misinformation on social media and its influence on public understanding and care of epilepsy [12].

There has been limited research into trends of discussions on health conditions on Reddit, as this social media platform has only recently gained popularity. The subreddit r/Epilepsy serves as an ideal target for research, as this subreddit is an active community of individuals sharing lived experiences, questions, and resources related to epilepsy. A study by Dahiya and Bagga in 2024 analyzed over 50,000 r/Epilepsy posts from 2021 to 2024 and identified key themes of depression, driving restrictions, job concerns, and pregnancy issues [13]. Building upon this work, which provided valuable insights into the themes of concerns and mental health overlap in r/Epilepsy postings, this study aims to delve deeper into the temporal dynamics and emotional tones throughout the r/Epilepsy community. Through analyzing subreddit posts from 1 December 2023 to 31 December 2024, this study aims to explore trends in the volume, timing, themes, emotional tone, and sentiment of posts, which have previously been unexamined, within this online epilepsy Reddit community. Understanding these patterns provides insight into how individuals with epilepsy engage with online communities over time, express emotional states, and seek informational or social support. Such insights can inform healthcare providers, patient advocacy organizations, and digital health researchers in designing interventions, educational resources, and support mechanisms that are responsive to real-world patient needs and experiences.

## 2. Methods

Posts were retrieved from the r/Epilepsy subreddit using Reddit’s Application Programming Interface (API) between 1 December 2023 and 31 December 2024. Data extraction was restricted to original submissions (excluding comments and replies) and to English-language posts at the time of collection. Language restrictions were applied to ensure compatibility with lexicon-based sentiment analysis and TF-IDF vectorization. Exclusion of replies was intentional to focus on content that motivated users to initiate a post rather than subsequent conversational exchanges. Metadata retrieved for each post included the post body text and timestamp. All data analyzed was publicly available and de-identified. No interaction with users occurred.

A total of 25,222 posts were retrieved. During preprocessing, 556 posts (2.20%) were excluded due to missing or empty textual content, and 6 posts (0.02%) were excluded due to invalid timestamps. No additional exclusions were required after preprocessing, as language and submission-type filters were applied during initial data extraction.

To explore trends in frequency of posts, timestamps for each post were converted to a standardized datetime format and categorized by day of the week, as well as by calendar month. Differences in posting frequency across days of the week and months were first evaluated using chi-square goodness-of-fit tests. To provide inferential support beyond descriptive statistics, Poisson regression models were subsequently fitted for daily and monthly post counts, modeling day of week and month as categorical predictors. Statistical significance was defined as *p* < 0.05. All statistical tests were performed using Python (version 3.13.0).

Sentiment analysis was conducted using two lexicon-based natural language processing tools. Emotional tone was determined via the TextBlob Python library (version 0.19.0). A polarity score (−1 to +1) was assigned to each post, with −1 representing an extremely negative emotional tone, 0 indicating a neutral emotional tone, and +1 representing an extremely positive emotional tone. Words such as “thankful” or “hopeful” contribute to a positive score, while words like “scared” or “miserable” contribute to a negative score. Scores closer to 0 may indicate a more neutral emotional tone, or a posting containing a combination of positive and negative remarks, averaging out to a more neutral score. VADER (Valence Aware Dictionary and Sentiment Reasoner) (NLTK version 3.9.1) was then used to generate compound sentiment scores for each post. VADER is optimized for short, informal text and is widely used for sentiment analysis on social media platforms [14]. Compound sentiment scores represent the overall sentiment of the posting, on a standardized scale from −1 (extremely negative sentiment) to +1 (extremely positive sentiment). Positive sentiment was defined as VADER compound scores ≥ 0.05, neutral sentiment with a compound score of −0.05 to 0.05, and a negative sentiment with a compound score of ≤−0.05. Agreement between VADER compound scores and TextBlob polarity scores was assessed using Pearson correlation.

To assess the robustness of automated sentiment classification in health-related discourse, a random subset of 120 posts was selected for manual sentiment validation. Posts were independently labeled as positive, neutral, or negative based on overall emotional tone. Manual sentiment labels were compared with VADER-derived classifications using Cohen’s kappa to evaluate concordance between automated and human sentiment assessments.

A KMeans clustering algorithm (scikit-learn version 1.6.1), an unsupervised learning algorithm, was used for thematic analysis with Python (version 3.13.0). Prior to analysis, extracted post data was processed by converting all text to lowercase and removing all punctuation, special characters, uniform resource locators (URLs), and numerical digits. Then, the post content was converted into numerical form using Term Frequency-Inverse Document Frequency (TF-IDF) vectorization. TF-IDF allows assessment of the frequency of terms appearing in post content for comparison [15]. A KMeans clustering algorithm was then applied to generate five clusters of similar posts, based on similar content patterns and vocabulary. A random state of 42 was used to ensure reproducibility of results. The number of clusters was selected based on exploratory inspection and silhouette analysis. Cluster stability was evaluated across multiple random initializations, and final cluster assignments were retained for subsequent human validation. Clusters dominated by removed or deleted content were excluded from thematic interpretation.

To validate machine-generated themes, a stratified random sample of 120 posts (40 per substantive cluster) was manually labeled by two reviewers using predefined thematic categories. Cluster-to-label correspondence was assessed using a majority-vote mapping approach. Inter-method agreement between KMeans cluster assignments and manual thematic labels was quantified using Cohen’s kappa.

Python (version 3.13.0) and Pandas (version 2.2.3) were used for data manipulation and statistical analysis. Matplotlib (version 3.10.0) was utilized for data visualization for trends. A minimal replication package including data cleaning scripts, analysis code, validation datasets, post identifier lists, and figure-generation scripts has been deposited in a public repository.

## 3. Results

### 3.1. Trends in Posting Frequency

**Figure 1:**
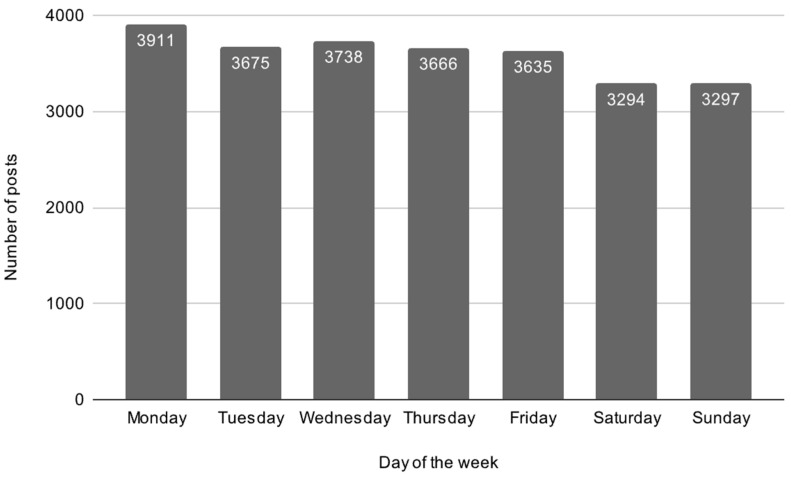
Number of posts submitted to r/Epilepsy across days of the week. Posting activity was highest on Monday (*n* = 3911) and lowest on the weekend (Saturday and Sunday). Values represent raw post counts aggregated over the sampled time period.

**Figure 2:**
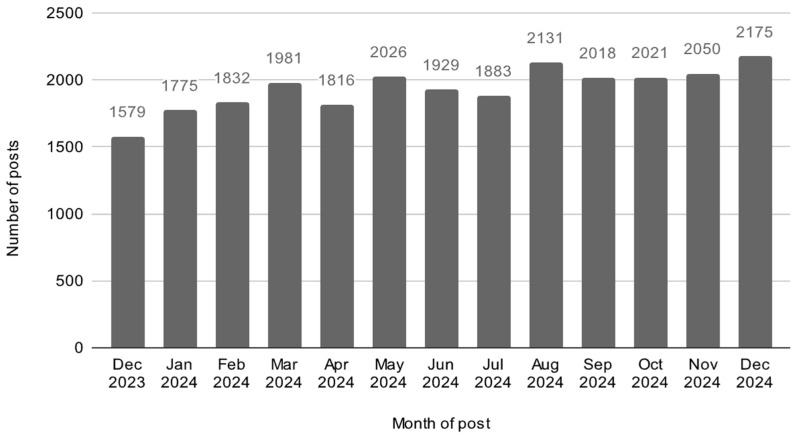
Monthly number of posts in r/Epilepsy from December 2023 to December 2024. Posting frequency steadily increased over the year, with the highest number of posts observed in December 2024 (*n* = 2175). The lowest post volume was in December 2023 (*n* = 1579).

A total of 25,222 posts were collected for trend analysis. Posting activity changed over time, with certain months and days seeing more posts than others. Peak posting volume was observed in the months of August and December, with a notable concentration of posts on Mondays (Figure 1 and Figure 2). A chi-square test revealed a statistically significant difference in post frequency by day of the week (χ^2^ = 86.75, *p* < 0.001), indicating that posts were not equally distributed across the week.

Poisson regression modeling further confirmed significantly higher posting frequency on Mondays compared to the reference day (*p* = 0.001), and significantly lower activity on Saturdays and Sundays (*p* < 0.001).

The highest number of posts occurred on Mondays, with a total number of 3911 posts (Figure 1). A chi-squared test showed significant differences in posts by month (χ^2^ = 152.26, *p* < 0.001), with the highest number of posts in December 2024 (2175) (Figure 2).

Month-level Poisson regression demonstrated a marked increase in posting frequency in December 2024 (*p* < 0.001), consistent with descriptive trends.

### 3.2. Sentiment and Emotional Tone Analysis

**Figure 3:**
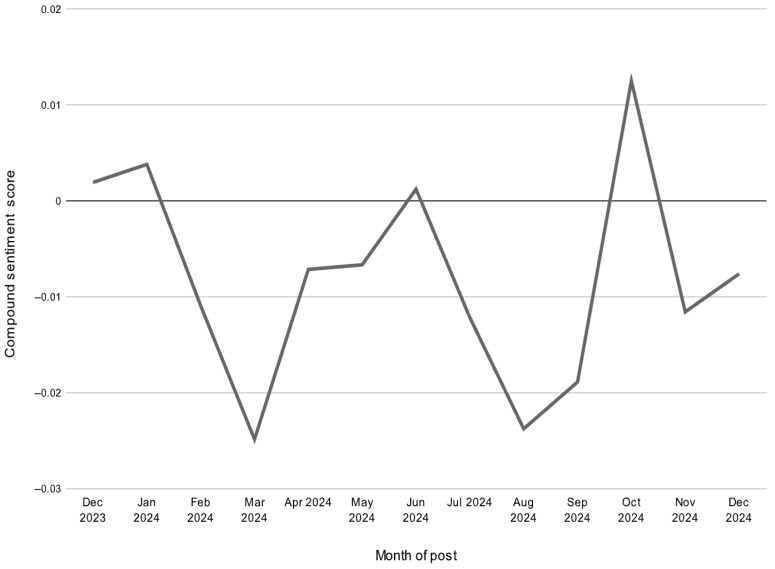
Monthly average compound sentiment scores of r/Epilepsy posts from December 2023 to December 2024. Sentiment fluctuated throughout the year, with notable dips in March and August 2024 and a peak in October 2024. Scores are calculated using the VADER sentiment analysis tool, where positive values represent more positive sentiment and negative values indicate more negative sentiment.

Sentiment analysis over time revealed notable shifts in user sentiments throughout the year. The periods of increased positive sentiments were mostly seen in January and October, while the more negative sentiments appeared during August and March (Figure 3). VADER compound sentiment scores and TextBlob polarity scores demonstrated moderate correlation (r = 0.393), supporting partial concordance between the two lexicon-based sentiment tools.

To assess the robustness of automated sentiment classification in health-related discourse, a random subset of 120 posts was manually annotated for sentiment. Comparison between VADER-derived sentiment classifications and human sentiment labels demonstrated moderate agreement (Cohen’s kappa = 0.52), indicating reasonable concordance while also highlighting the limitations of lexicon-based tools in capturing nuanced or context-dependent emotional expression.

### 3.3. Thematic Analysis

To explore the recurring topics and underlying themes observed in the dataset, a KMeans clustering analysis was performed. The analysis uncovered five main thematic clusters that capture the core focus areas of user discussions: treatment experiences, community engagement, personal experiences, solidarity, and gratitude toward the subreddit community. These themes reflect a blend of practical support, emotional sharing, and community cohesion that characterize user interactions within the Reddit platform.

Cluster separation was modest (mean silhouette score = 0.119) but highly stable across ten random initializations (standard deviation < 0.001), indicating a reproducible thematic structure.

To validate machine-generated clusters against human interpretation, a stratified random sample of 120 posts (40 posts per substantive cluster) was manually labeled. Using a majority-vote mapping approach to align cluster labels with human-coded themes, the five-category comparison yielded moderate agreement between KMeans clusters and manual thematic labels (Cohen’s kappa = 0.537).

Thematically related clusters were further collapsed into three broader categories: engagement/support, experience, and gratitude. When condensed into these categories, agreement increased substantially, yielding strong concordance between machine-generated clusters and human labels (Cohen’s kappa = 0.714).

These validation results support the interpretability and reproducibility of the identified thematic structure while acknowledging the inherent limitations of unsupervised topic modeling in complex, patient-generated discourse.

## 4. Discussion

This study analyzed over 25,000 posts from the r/Epilepsy subreddit, revealing patterns in posting frequency, emotional sentiment, and thematic content. Our findings highlight how Reddit functions as a dynamic space for individuals with epilepsy or connections to epilepsy to share experiences, seek support, and discuss concerns regarding treatment.

Subreddit postings peaked in December 2024 and were significantly higher than those seen in the previous year in December 2023. Poisson regression modeling confirmed statistically significant day-of-week and month effects, providing inferential support for these observed temporal trends. Notably, Epilepsy Awareness Month is in November of each year, with many foundations and patient advocacy groups setting up events to spread awareness and engagement. The observed increase in r/Epilepsy postings over time may be consistent with heightened awareness activity and broader engagement with epilepsy-related topics. However, our analyses did not directly test the impact of specific campaigns or external events, and these associations should be interpreted as hypotheses rather than causal conclusions. Similar seasonal posting surges have been documented in other health-related social media forums, suggesting that awareness efforts and public visibility may coincide with increased online engagement [16]. This rise is also occurring during a period of increased public attention on Reddit following its Initial Public Offering (IPO) [4], as well as a period characterized by ongoing global growth in social media use for health-related information [2]. Future research incorporating event-based modeling, placebo time points, or external reference series could more rigorously evaluate whether awareness campaigns or platform-level shifts contribute to observed posting trends.

As prior work has also suggested, the posting patterns may reflect individuals seeking peer support, sharing experiences, or researching to optimize their care following epilepsy-related events or awareness initiatives [5,8]. The Dahiya & Bagga 2024 study reported increased posting following International Epilepsy Day and Purple Day, which occurs annually on 26 March [13]. While our findings align directionally with this literature, the present study remains observational and cannot determine whether awareness campaigns directly influenced user behavior. Instead, results suggest temporal associations that warrant more targeted investigation [17,18].

Subreddit peak postings were on Mondays. This pattern may reflect users seeking support or sharing experiences at the start of the week, possibly after weekend health events or personal reflections. Alternatively, this pattern may simply reflect broader weekday engagement behaviors common across online communities. Similar early-week activity patterns have been noted in other chronic illness community forums [19]. Although we hypothesize that Monday peaks could represent structured community check-ins or increased coping-related posting, causal mechanisms cannot be inferred from aggregate posting data alone.

The emotional tone of posts on r/Epilepsy shifted throughout the year, with more positive sentiment seen in January and October of 2024, and more negative sentiment in March and August. These trends may reflect seasonal mood variations, shifts in individual life stressors, treatment-related experiences, or even broader external factors, such as medication access or health policy changes. Notably, October coincides with National Epilepsy Awareness Month, which may contribute to shifts in tone; however, this interpretation remains speculative. Importantly, average sentiment values remained near neutral overall, suggesting a complex mixture of informational, supportive, and emotionally varied discourse. Manual validation demonstrated moderate agreement between automated sentiment classification and human ratings, supporting reasonable concordance while underscoring the limitations of lexicon-based tools in capturing nuanced health-related language. Lexicon-based tools may not fully capture sarcasm, ambivalence, or medically contextual language.

While other studies have focused mainly on misinformation or specific themes in online epilepsy discussions, our approach adds another layer by capturing how people are feeling when they post. This is often an aspect of social media that is harder to quantify, but a very significant consideration in assessing patient experience and identifying areas of support needs. Our approach integrates temporal modeling, sentiment analysis, and thematic clustering to provide a multidimensional overview of community discourse. Thematic validation demonstrated moderate to strong agreement with human coding, supporting the interpretability of the identified themes while acknowledging the abstraction inherent in unsupervised modeling.

Sentiment analysis of patient-generated content has increasingly been recognized as a scalable proxy for real-time mood and emotional state, supplementing traditional qualitative research [20,21]. However, sentiment signals should not be interpreted as direct measures of psychological state. Instead, they represent aggregated linguistic patterns that may approximate shifts in tone over time. These emotional trends could be used by healthcare providers, advocacy groups, and public health organizations to tailor support efforts during periods of heightened need, for example, by increasing access to online resources, encouraging peer support, or offering telehealth during months when sentiment is more negative. At the same time, our findings do not establish that targeted interventions would directly alter online behavior or emotional tone.

Thematic clustering identified five main themes throughout the subreddit posts: treatment experiences, community engagement, personal stories, solidarity, and appreciation for the subreddit itself. These themes share many similarities with those identified in previous studies, such as psychological burden and treatment navigation. However, our analysis further highlights the importance of gratitude and peer support, specifically in the Reddit community, reflecting the emotional value of online r/Epilepsy community participation. The high volume of posts focused on treatment experiences may reflect users’ tendency to turn to the subreddit for guidance on medication decisions, share side effects, or inquire about others’ experiences when they do not have easy access to specialized epilepsy care. This finding aligns with research showing that online patient communities frequently serve as critical sources for experiential knowledge and emotional validation, especially when traditional healthcare access is limited or nonexistent [22,23].

Collectively, these findings extend existing knowledge by providing real-time, patient-centered insights that can inform healthcare providers, policymakers, and advocacy groups. Awareness of temporal trends in posting behavior and sentiment could help guide the timing of targeted interventions, such as offering online mental health resources during periods of lower sentiment (e.g., March and August) or synchronizing informational campaigns with high-traffic periods. The prominent role of community gratitude and solidarity points to the emotional value patients derive from peer support, suggesting that clinicians might consider referring patients to reputable online communities alongside traditional resources [8,13].

In recent years, social media platforms have become vital spaces where people not only connect socially but also share personal experiences about their health. For individuals with chronic conditions like epilepsy, online communities offer a unique opportunity to express concerns, seek advice, and find support from peers who truly understand and connect to their journey [10,11].

Our combined use of large-scale sentiment analysis and thematic clustering provides a more comprehensive understanding of the epilepsy community’s lived experience on social media than prior studies, which often focus on single dimensions such as misinformation or isolated themes. While much existing research on epilepsy tends to focus on clinical or medical contexts, there has been limited attention given to conversations driven by patients online. This multidimensional approach reveals not only what topics users discuss but also how they feel, emphasizing the emotional complexity and everyday challenges that may be overlooked in a traditional care setting. By tapping into these online conversations, healthcare providers and advocacy groups can gain a clearer picture of what’s important to patients outside the clinical setting. This real-world insight can highlight gaps in care, common frustrations, and emotional struggles that might not be captured during office visits. Moreover, as patients increasingly seek information and community online, there is a growing opportunity for clinicians to engage with these platforms more directly. Sharing reliable, up-to-date medical information on Reddit and similar forums could help counteract misinformation and empower patients with accurate knowledge. This is especially important for those who have limited access to specialized care or who feel isolated in their condition [2].

## 5. Limitations

This study has several limitations that should be acknowledged. First, our data source was limited to posts from the r/Epilepsy subreddit, which may not fully represent the diversity of people living with epilepsy or those affected by it. Additionally, user demographics such as age, gender, or geographic location are not available from the posts due to Reddit’s anonymous blogging nature, limiting our ability to analyze how these factors might influence discussions and sentiments. Additionally, due to this anonymity, we cannot confirm whether users posting in the r/Epilepsy subreddit have a formal diagnosis of epilepsy.

Another important limitation involves the tools used for sentiment analysis. While VADER and TextBlob are well-established and widely used, they may not capture the full complexity of medical conversations, especially when users describe ambivalent emotions, sarcasm, or context-dependent experiences related to epilepsy. Although moderate concordance between automated sentiment classifications and humans was observed, lexicon-based approaches are not domain-adapted to epilepsy-specific discourse and may under- or overestimate sentiment in certain contexts.

Importantly, this study analyzed only English-language original submissions. Language restrictions were applied because both TF-IDF vectorization and lexicon-based sentiment tools rely on English-language processing, and inclusion of multilingual posts would have required separate validated analytic pipelines. Replies and comment threads were excluded to focus on the motivations prompting users to initiate posts rather than subsequent conversational exchanges. While these decisions enhanced analytic consistency and interpretability, they may introduce selection bias and limit representation of multilingual users and interactive discussion dynamics within the broader subreddit ecosystem.

Additionally, we did not evaluate the accuracy or clinical validity of medical information shared in posts. Online health communities can contain misinformation, anecdotal advice, or non-evidence-based recommendations, which may influence patient decision-making. Although this study did not directly assess misinformation prevalence, future research should examine both the reliability of shared content and potential mitigation strategies, such as collaboration between clinicians and community moderators or the promotion of verified resources within digital spaces.

Finally, findings from this specific online community may not generalize to all individuals with epilepsy, especially those who do not engage with social media or prefer to participate in different digital platforms. Future research could expand analyses to additional subreddits, multilingual datasets, or cross-platform comparisons to better understand the broader digital landscape of epilepsy-related discourse.

## 6. Conclusions

This study highlights Reddit as a valuable platform for individuals to share and discuss their experiences with epilepsy. By analyzing posts throughout the year, we observed clear patterns in posting behavior, as well as meaningful trends in sentiment and emotional tone. Sentiment analysis helped identify emotional highs and lows throughout the year, while thematic analysis revealed common topics such as treatment experiences, personal stories, community support, and gratitude. Together, these findings provide a deeper understanding of the concerns and experiences of people affected by epilepsy. For healthcare professionals and researchers, social media like this can offer real-time insights into patient needs, emotional well-being, and emerging topics of interest. This kind of analysis can support more responsive, patient-centered care and inform future research efforts aimed at improving the lives of those living with epilepsy.

Building on this study, future research could explore epilepsy discussions across multiple social media platforms, such as X (formerly Twitter) and Instagram, to compare content, user engagement, and sentiment trends. This broader approach could provide a more comprehensive picture of how people with epilepsy communicate and support each other online. Further investigation into specific epilepsy-related concerns, such as treatment side effects, seizure triggers, or access to care, would be valuable to better understand patient priorities and unmet needs. Additionally, there is a need to improve sentiment analysis methods for medical discussions. Developing tools that can better interpret complex emotions and medical terminology could enhance the accuracy and usefulness of online patient sentiment studies. Incorporating assessments of information accuracy and identifying sources of misinformation on social media platforms should also be a priority, helping clinicians and advocacy groups better address false or harmful content.

## Data Availability

The data presented in this study are available on request from the corresponding author. A minimal replication package including data extraction and cleaning scripts, post ID lists, analysis notebooks, and code to regenerate all figures has been deposited in a public repository with a persistent identifier (DOI: 10.5281/zenodo.18664562).
